# The Use of Inflammatory Markers to Rule Out Acute Appendicitis in Pediatrics

**DOI:** 10.7759/cureus.31374

**Published:** 2022-11-11

**Authors:** Mohammad Halawani, Ahmed Mnofala, Hatoon Hakeem, Ahmed Othman, Mahmoud Halawani, Abdulhadi Tashkandi

**Affiliations:** 1 Emergency Department, Prince Mohammed Bin Abdulaziz Hospital, Madinah, SAU; 2 Emergency Department, King Abdulaziz Medical City, Jeddah, SAU

**Keywords:** white blood count, crp, infection, inflammatory, appendicitis

## Abstract

Background

Acute appendicitis is the most common abdominal surgical emergency in pediatric patients. The diagnosis of acute appendicitis in pediatrics is challenging and requires an accurate physical examination, laboratory study, and imaging. The aim of this study was to determine the benefits of using three inflammatory markers, white blood count (WBC), neutrophils percent (NE%), and C-reactive protein (CRP), in ruling out appendicitis in pediatric patients.

Methods

A retrospective study was conducted of 152 pediatric patients aged between 6 months and 14 years presenting to the emergency department between January 2018 and December 2020, with the diagnosis of appendicitis as the primary physician’s main diagnosis. Demographic information and clinical data were extracted from the medical file for each patient.

Results

Out of the 152 patients included, 68 (44.7%) were female and 84 (55.3%) were male, with median age was 8.1 years. Thirty-six (23.7%) had acute appendicitis confirmed by histopathology. Of these 36 patients, only two patients (5.6%) had all inflammatory markers within normal limits.

Conclusion

Although raised inflammatory markers may help diagnose acute appendicitis, their role in ruling it out remains limited.

## Introduction

Appendicitis represents the most common abdominal surgical emergency in the pediatric age group [1]. Despite being a relatively common condition, the diagnosis of appendicitis in children can prove challenging in many cases [1]. The utility of history and physical examination is limited in young children who lack the verbal skills to provide an accurate description of their symptoms [2]. In addition, the classic symptoms for detecting appendicitis in adults are not encountered in all pediatrics; in such young patients, the abdominal examination is more challenging due to a lack of cooperation and appropriate expressions. Among younger patients, the incidence of complicated appendicitis is common. Due to its complexity, missed appendicitis is a common cause of lawsuits in the emergency department (ED). Luminal occlusion due to various etiologies and bacterial infections of the appendix is typically held responsible for the pathogenesis of this disease, which is mostly seen in adolescents [3].

Although inflammatory markers are still being studied in the presence of appendicitis, a definite conclusion has not been reached yet. The laboratory evaluation of patients with suspected appendicitis, including white blood cell count (WBC), C-reactive protein (CRP), and other serum markers, either alone or in combination, can be helpful, but the reported sensitivities and specificities for these markers are highly variable and not independently reliable for accurately excluding or confirming the diagnosis [2]. We still rely on imaging for the diagnosis of appendicitis in pediatrics, with the ultrasound (US) as the first modality, with its relatively low sensitivity and specificity [4]. Computerized tomography (CT) is, however, sometimes necessary to rule out appendicitis.

## Materials and methods

This retrospective observational study was conducted in Prince Mohammed bin Abdulaziz Hospital, Ministry of National Guard for Health Affairs, Madinah, Kingdom of Saudi Arabia. Data were collected via electronic systems for all pediatric patients presenting to the ED with suspicion of acute appendicitis from 2018 to 2020. We primarily investigated the three inflammatory markers (white blood count (WBC), neutrophils percent (NE%), and C-reactive protein (CRP)) in pediatric patients with suspicion of acute appendicitis as the primary diagnosis based on history and clinical evaluation.

Inclusion criteria

All patients <14 years presented to the emergency department with suspected acute appendicitis and had inflammatory blood test results available in the hospital medical records and underwent either US or CT imaging or both.

Exclusion criteria

We excluded any patient missing one or more of the inflammatory markers.

A total of 252 patients’ medical records were retrieved. Of these, 152 patients fit the inclusion criteria. There were 68 female patients and 84 male patients mainly between the ages of 6 years and 12 years as shown in Table 1. The following laboratory reference cutoffs were used: WBC ˃ 12,000, NE% ˃ 75%, and CRP ˃ 5 mg/L. We conducted this study primarily to determine the possibility of ruling out acute appendicitis in patients with abdominal pain when all three inflammatory markers results were negative. As such, we examined all the inflammatory markers results combined and looked into the patients with positive appendicitis with no abnormality in those markers.

**Table 1:** Demographic data

Demographic data	frequency (%)
Gender	
Female	68 ( 44.7%)
Male	84 (55.3%)
Age	
6 months	1(0.7%)
22 months	1(0.7%)
2 yrs.	4 (2.6%)
3 yrs.	5 (3.3%)
4 yrs.	7 (4.6%)
5 yrs.	5 (3.3%)
6 yrs.	18 (11.8%)
7 yrs.	25 (16.3%)
8 yrs.	19 (12.4%)
9 yrs.	14 (9.2%)
10 yrs.	20 (13.1%)
11 yrs.	19 (12.4%)
12 yrs.	12 (7.8%)
13 yrs.	2 (1.3%)

## Results

Of the 252 patients, 152 met our inclusion criteria. Of these patients, 68 (44.7%) were female and 84 (54.9%) were male. Among all patients, 36 had confirmed appendicitis by radiological studies. All positive cases went for appendectomy; all 36 patients had an inflamed appendix on the histopathology report. Of the 36 positive, 14 (9.2%) were female and 22 (14.4%) were male (Table 2). Out of the 36 positive cases, only two had all inflammatory markers within normal limits (Table 3). Most cases were between the age of 7 and 11 years (Table 4).

**Table 2:** Association between the gender and the radiological findings of the patient

Association between gender and positive appendicitis			
Gender			Total
	Negative result	Positive result	
Female	54 (35.5%)	14 (9.2%)	68 ( 44.7%)
Male	62 (40.78%)	22 (14.4%)	84 (54.9%)
Total	116 (76.3%)	36 (23.7%)	152 (100%)

**Table 3:** Association between CRP, WBC, and NE% and the diagnosis of acute appendicitis WBC: white blood count; NE%: neutrophils percent; CRP: C-reactive protein

Association between CRP, WBC, and NE% and the diagnosis of acute appendicitis			
Lab test	Positive appendicitis	Negative appendicitis	Total
Positive test	34	79	113
Negative test	2	37	39
Total	36	116	152

**Table 4:** Association between age and the radiological findings of the patient

Association between the age and the radiological finding of the patient			
Age	Radiological finding		Total
	Negative result	Positive result	
6 months	1 (0.7%)	0	1 (0.7%)
22 month	1 (0.7%)	0	1 (0.7%)
2 yrs.	3 (2%)	1(0.7%)	4 (2.6%)
3 yrs.	5 (3.3%)	0	5 (3.3%)
4 yrs.	5 (3.3%)	2 (1.3%)	7 (4.6%)
5 yrs.	4 (2.6%)	1 (0.7%)	5 (3.3%)
6 yrs.	16 (10.5%)	2 (1.3%)	18 (11.8%)
7 yrs.	21 (13.7%)	4 (2.6%)	25 (16.3%)
8 yrs.	13 (8.5%)	6 (3.9%)	19 (12.4%)
9 yrs.	9 (5.9%)	5 (3.3%)	14 (9.2%)
10 yrs.	14 (9.2%)	6 (3.9%)	20 (13.1%)
11 yrs.	14 (9.2%)	5 (3.3%)	19 (12.4%)
12 yrs.	9 (5.9%)	3 (2%)	12 (7.8%)
13 yrs.	1 (0.7%)	1 (0.7%)	2 (1.3%)
Total	116 (76.3%)	36 (23.7%)	152 (100%)

CRP was noted to be high in 28 (18.3%) patients with appendicitis as shown in Table 5. This gives it more sensitivity and specificity than WBC and NE%, which was noted to be elevated in only 25 patients (16.3%), as shown in Table 6-7. sensitivity, specificity, positive predictive values, and negative predictive values are shown in Table 8.

**Table 5:** Association between the inflammatory marker CRP (more than 5mg/l) and the radiological finding of the patient CRP: C-reactive protein

Association between the inflammatory marker CRP (more than 5 mg/l) and the radiological findings of the patient			
CRP more than 5mg/l	Radiological finding		Total
	Negative result	Positive result	
Negative	53 (34.8%)	8 (5.2%)	61 (40.1%)
Positive	63 (41.2%)	28 (18.3%)	91 (59.5%)
Total	116 (76.3%)	36 (23.7%)	152 (100%)

**Table 6:** Association between the inflammatory marker WBCs (above 12K) and the radiological finding of the patient

Association between the inflammatory marker WBCs (above 12K) and the radiological finding of the patient			
(WBCs above 12K)	Radiological finding		Total
	Negative result	Positive result	
Negative	73 (48 %)	11 (7.2%)	84 (55.2%)
Positive	43 (28.1%)	25 (16.3%)	68 (44.4%)
Total	116 (76.3%)	36 (23.7%)	152 (100%)

**Table 7:** Association between the inflammatory marker neutrophil (>75%) and the radiological findings of the patient

Association between the inflammatory marker neutrophil (>75%) and the radiological finding of the patient			
Shift to left Neutrophil >75%	Radiological finding		Total
	Negative result	Positive result	
Negative	65 (42.7%)	11 (7.2%)	76 (50 %)
Positive	51 (33.3%)	25 (16.3%)	76 (49.7%)
Total	116 (76.3%)	36 (23.7%)	152 (100%)

**Table 8:** Inflammatory markers with their sensitivity, specificity, PPV, and NPV PPV: positive predictive value; NPV: negative predictive value

	WBC ˃ 12,000	CRP ˃ 5 mg/L	NE ˃ 75%	All markers elevated
Sensitivity %	69	78	69	94
Specificity %	62	46	56	31
PPV %	36	31	33	30
NPV %	87	87	86	94

We noted that with all three markers combined, the sensitivity increased to 94% with a specificity of 31%, a positive predictive value of 30%, and a negative predictive value of 94%. This means that the probability of the disease not to present when the tests are negative is 94%.

## Discussion

Acute appendicitis is characterized by the development of inflammation at a local level, followed by a more generalized inflammatory response [5]. The rationale of laboratory tests in the diagnosis of acute appendicitis is based on the possibility of detecting signs of systemic inflammation with a diagnostic tool that is widely available, easy to use, minimally invasive, has limited costs, and can be repeated if necessary [5]. The inflammatory markers in diagnosing acute appendicitis have a wide variety in sensitivity and specificity. A study conducted by JJY Kim shows that WBC has lower sensitivity than CRP [6], which goes with our finding. Many other studies show the opposite, with WBC having higher sensitivity and specificity [7-9].

It is difficult to rely only on inflammatory markers alone to diagnose acute appendicitis in pediatric patients although the presence of negative inflammatory markers makes the probability low [10].

In our study, we found that the negative predictive value was reaching 94% with only two patients having normal inflammatory markers diagnosed as appendicitis. In another study by Sengupta et al., it was found that normal WBC and CRP have a 100% negative predictive value, which is against our findings [9]. Although they didn’t look at the NE% in the study, in our study, one patient had appendicitis with only elevated NE%.

The meta-analysis by Andersson found that where all markers of inflammation were normal and the negative likelihood ratio is less than 0.10, but not zero, indicating that appendicitis is still possible with normal inflammatory markers [11]. Another study by Shaw found similar results to our study [8]. This makes the role of inflammatory markers limited when ruling out acute appendicitis but the appropriate use of these markers combined can help in supporting the diagnosis.

There are multiple new studies looking at other inflammatory markers to help diagnose acute appendicitis; for example, the study by Bozlu looked at the red blood cell distribution width in combination with WBC and CRP with no clear role in ruling out appendicitis [12]. Another study by Nissen looked into the lymphocyte-to-monocyte ratio to differentiate complications with non-complicated appendicitis; their role in ruling out appendicitis is not yet clear [13]. Looking into the scoring system as the solo method for ruling out appendicitis, multiple studies were done discussing score validation. The pediatric appendicitis score (PAS) is one of the most commonly used and has good validation. However, if we used PAS alone, many patients with acute appendicitis will be missed and unsafely will be discharged home [14,15]. A new, validated scoring system Pediatric Appendicitis Risk Calculator (pARC) shows no missed cases in the low-risk group [16], but before fully adopting pARC in our practice, more studies should focus on its ability to rule out appendicitis.

The limitations we faced included the number of positive cases collected during the study period, which might be related to it being a single-center study with an average of 70,000 patient visits per year or to the unavailability of pediatric surgeons around the clock so patients with suspected appendicitis might have been triaged to another health care facility.

More studies

We believe more studies should be conducted in this regard, with larger sample size, the examination of symptom onset, and the possibility of combining clinical decision rules with inflammatory markers.

Final thoughts

As emergency departments are getting busier and the load to emergency departments is increasing, we are seeking solutions to decrease the ED stay without compromising patient safety. With this result, we think it is still unsafe to discharge patients to their homes relying only on inflammatory markers.

## Conclusions

The diagnosis of appendicitis remains multifactorial and relies largely on clinical assessment rather than lab tests. The use of inflammatory markers can help in diagnosing appendicitis, but its role in ruling it out remains limited.

This study shows that acute appendicitis can occur with normal inflammatory markers; with this finding, we should be focusing more on the clinical assessment of patients with the proper use of adjuncts to help in diagnosing acute appendicitis.
